# Structurally Colored Cellulose Nanocrystal Films as Transreflective Radiative Coolers

**DOI:** 10.1021/acsnano.1c10959

**Published:** 2022-06-06

**Authors:** Ravi Shanker, Prasaanth Ravi Anusuyadevi, Sampath Gamage, Tomas Hallberg, Hans Kariis, Debashree Banerjee, Anna J. Svagan, Magnus P. Jonsson

**Affiliations:** † Laboratory of Organic Electronics, Department of Science and Technology, Linköping University, SE-601 74 Norrköping, Sweden; ‡ Wallenberg Wood Science Center, Linköping University, SE-601 74 Norrköping, Sweden; § Royal Institute of Technology (KTH), Dept. of Fibre and Polymer Technology, SE-100 44 Stockholm, Sweden; # FOI-Swedish Defense Research Agency, Department of Electro-Optical systems, 583 30 Linköping, Sweden; ∥ Department of Chemical Engineering, M S Ramaiah Institute of Technology, 560054 Bangalore, Karnataka India

**Keywords:** passive radiative cooling, cellulose nanocrystals, structural colors, self-assembly, thermal radiation, atmospheric transparency window

## Abstract

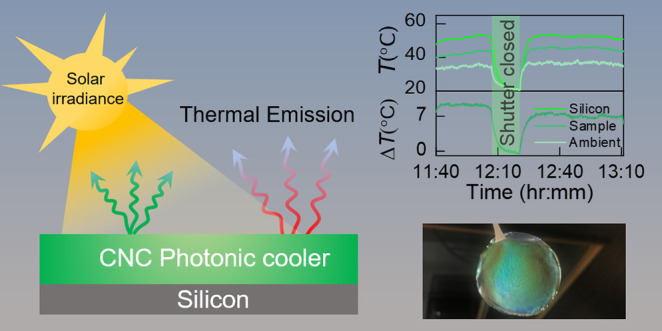

Radiative cooling forms an emerging direction in which objects are passively cooled via thermal radiation to cold space. Cooling materials should provide high thermal emissivity (infrared absorptance) and low solar absorptance, making cellulose an ideal and sustainable candidate. Broadband solar-reflective or transparent coolers are not the only systems of interest, but also more pleasingly looking colored systems. However, solutions based on wavelength-selective absorption generate not only color but also heat and thereby counteract the cooling function. Intended as coatings for solar cells, we demonstrate a transreflective cellulose material with minimal solar absorption that generates color by wavelength-selective reflection, while it transmits other parts of the solar spectrum. Our solution takes advantage of the ability of cellulose nanocrystals to self-assemble into helical periodic structures, providing nonabsorptive films with structurally colored reflection. Application of violet-blue, green, and red cellulose films on silicon substrates reduced the temperature by up to 9 °C under solar illumination, as result of a combination of radiative cooling and reduced solar absorption due to the wavelength-selective reflection by the colored coating. The present work establishes self-assembled cellulose nanocrystal photonic films as a scalable photonic platform for colored radiative cooling.

The concept of passive radiative cooling (PRC) takes advantage of the atmosphere being largely transparent to light in the spectral range between 8 and 13 μm, which nicely coincides in wavelength with thermal radiation from objects at room temperature.^1−6^ Objects on earth can therefore radiatively transfer thermal energy into cold space, with less thermal energy being radiated back from the atmosphere. Combined with efficient suppression of solar absorption, PRC can passively reduce the temperature of objects to below that of the ambient even in daytime under solar illumination.^7^ Suitable materials for PRC should provide not only high thermal emissivity (i.e., strong infrared absorption) but also minimal absorption in the solar spectrum. This has led to an increasing interest in cellulose-based PRC.^8,9^ Cellulose is an abundant sustainable material with inherently strong thermal emissivity due to molecular resonances in the relevant far-infrared range. Furthermore, cellulose shows only minimal absorption of visible light. By controlling its microstructure and/or embedding light-scattering microparticles, it is possible to make cellulose materials that are either transparent, translucent or reflective, while visible absorption remains minimal.^10^ Scalable cellulose-based PRC systems were demonstrated both for reflective and transparent systems,^11^ with the latter intended for window applications or as coatings on photovoltaic cells and other optoelectronic devices.^12,13^

Besides completely white (reflective) and transparent systems, researchers are exploring ways to improve the appearance of PRC systems by introducing coloration.^14^ Colored passive radiative coolers (ColPRCs) will substantially expand the spectrum of potential applications in industries such as clothing, buildings, energy harvesting, and automobiles.^15−17^ ColPRC has been demonstrated using optically resonant structures^18^ or inorganic pigments and/or dyes to generate colors by wavelength-selective visible absorption.^15,19−21^ This absorption inevitably causes heat that counteracts the cooling process. Therefore, simultaneously achieving color and effective PRC remains a challenge. Interestingly, transparent ColPRC systems can circumvent this issue using wavelength-selective reflection/transmission with maintained low solar absorption.^22^ Aiming at sustainable scalable systems for such transreflective ColPRC, we turn to cellulose in the form of nanocrystals (CNCs), which can be derived from a number of different biomass resources, including agriculture and forestry residues.^23−25^ Interestingly, CNCs can self-assemble at large scale into chiral films with structural reflective colors.^26−31^ We show that such photonic CNC films can simultaneously provide desired reflective colors, low solar absorption, and strong thermal emissivity. This makes them interesting as a sustainable solution for transreflective ColPRC. We investigate the colored CNC films as thin-film coatings on silicon samples and show that they can provide both coloration and reduction in temperature by around 9 °C when exposed to solar light and the clear sky, as a result from both PRC and reduced solar absorption. The results show promise for cellulose structural systems for enabling the next-generation of scalable and sustainable ColPRCs.

## Results

**Figure 1:**
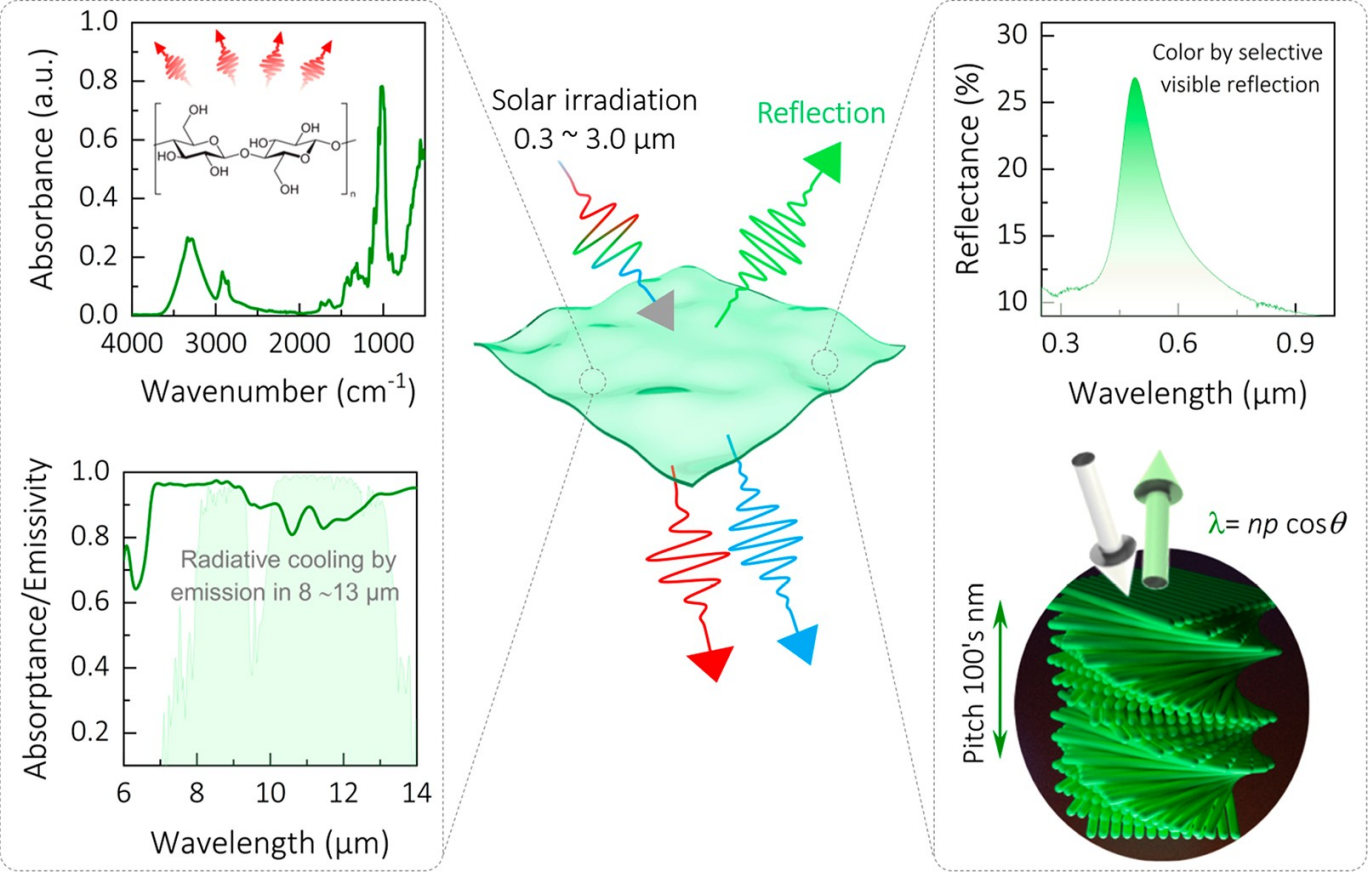
Concept of the cellulose ColPRC. (Left) Infrared absorbance (top) and thermal emissivity/absorptance (bottom) of a typical cellulose CNC ColPRC thin film. The inset in the top panel shows the molecular structure of cellulose. The shaded background spectrum in the lower panel presents the realistic atmospheric transmittance model. (Center) The schematic illustration in the center depicts the interaction of solar irradiation with the photonic CNC film. (Right) Selective reflection in the visible region generates structural color (top), with the color being determined by the chiral pitch of the CNC film (bottom).

Figure 1 depicts the main concept of CNC ColPRC, with the left panel indicating the strong infrared absorption and thermal emissivity of a typical CNC ColPRC film (processing conditions and color tuning is described below). The top left panel shows the Fourier transform infrared (FTIR) absorbance of a green ColPRC film, with contribution mainly from OH (3400 cm^–1^), C–H (3000–2700 cm^–1^), C–O, and C–O–C stretching or bending vibrations between 770 and 1350 cm^–1^ along with strong absorbance at ∼1050 cm^–1^ due to C–O.^10,32^ In the visible, the same photonic film generates structural coloration via wavelength-selective reflection, as shown in the top right panel by a clear reflectance peak. The structural color originates from the CNCs being arranged in twisted layers (depicted in the bottom right panel), leading to a photonic bandgap (and color) that depends on the pitch of the system.^8,10,26,27,33^

**Figure 2:**
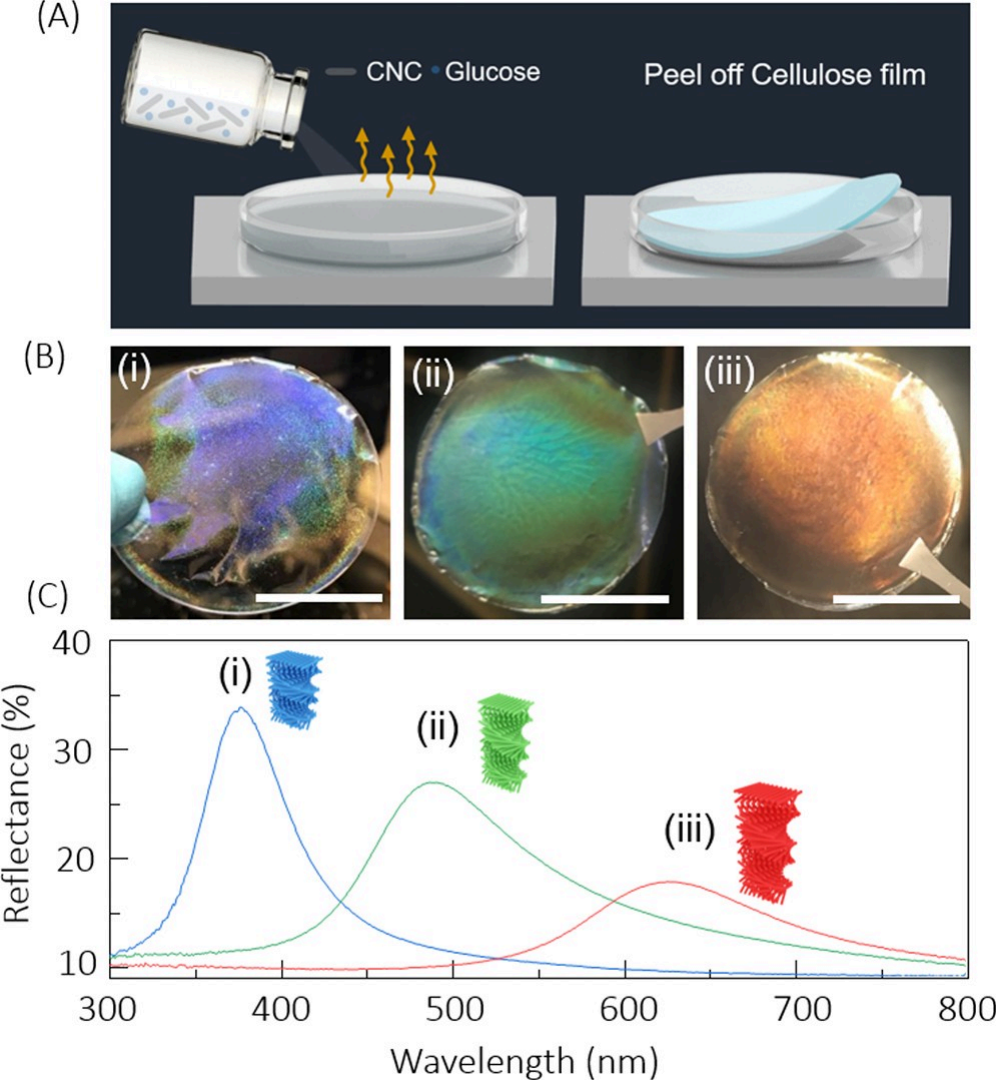
Self-assembly of CNC/GLU films with structural coloration. (A) Schematic illustration of the process of making self-assembled CNC ColPRC films. (B) Digital photographs of centimeter-sized ColPRCs producing (i) violet-blue , (ii) green, and (iii) red structural colors. The scale bars are 2.5 cm. (C) Reflectance spectra of the CNC ColPRC films, showing clear peaks that redshift with increasing GLU content. The insets show (false colored) schematics of the chiral nematic CNC structure, with corresponding increasing helical pitch.

Figure 2a shows a schematic illustration of the self-assembly process of CNCs into thin ColPRCs films. The starting aqueous CNC suspension (the preparation of CNCs is explained in the Methods section) was placed in a Petri dish and left drying with an open lid at ambient conditions, resulting in self-assembled colored thin films of cellulose nanocrystals. We prepared differently colored ColPRCs using different weight percentages (wt %) of glucose (GLU) content. Figure 2b shows digital photographs of CNCs with CNC/GLU content of (i) 66/34, (ii) 52/48, and (iii) 39/61. In agreement with our previous work,^30^ the photonic films exhibited bright colors under normal diffuse ambient light, with reflected colors gradually shifting from violet-blue to red with increasing GLU content (Figure 2b). Figure 2c shows the corresponding reflection spectra of the same samples, revealing clear peaks at 374 nm (i, violet-blue), 487 nm (ii, green), and 626 nm (iii, red). The reason for the red-shift with increasing GLU content is that the presence of the GLU increases the chiral nematic pitch (*P*, defined as the distance required for the CNC assembly to make 360° rotation) by intercalating the helical stacked CNC layers and increasing the distance between these layers (qualitatively illustrated in the insets in Figure 2c). In turn, the wavelength-selective reflection originates from Bragg diffraction according to^29^
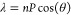
1where *n* is the mean refractive index, θ is the angle of incidence (with 0° being normal incidence), and *P* is the pitch of the helical structure as defined above. Notably, the helicity of the structure results in preferential reflection of left-handed circularly polarized light, as previously utilized in various applications.^29^ The increasing bandwidth and lower reflectivity with increasing GLU content are likely due to the polydomains with wide effective distribution of chiral pitches and/or tilted helical axes.^31^ Perpendicular cross-section SEM images of fractured films (Figure S1) reveal the internal cholesteric arrangement of ColPRC samples. The micrographs indicate layers that are stacked predominantly parallel to each other, while higher magnification imaging gives an indication of pitch length and preferential orientation of CNCs.

**Figure 3:**
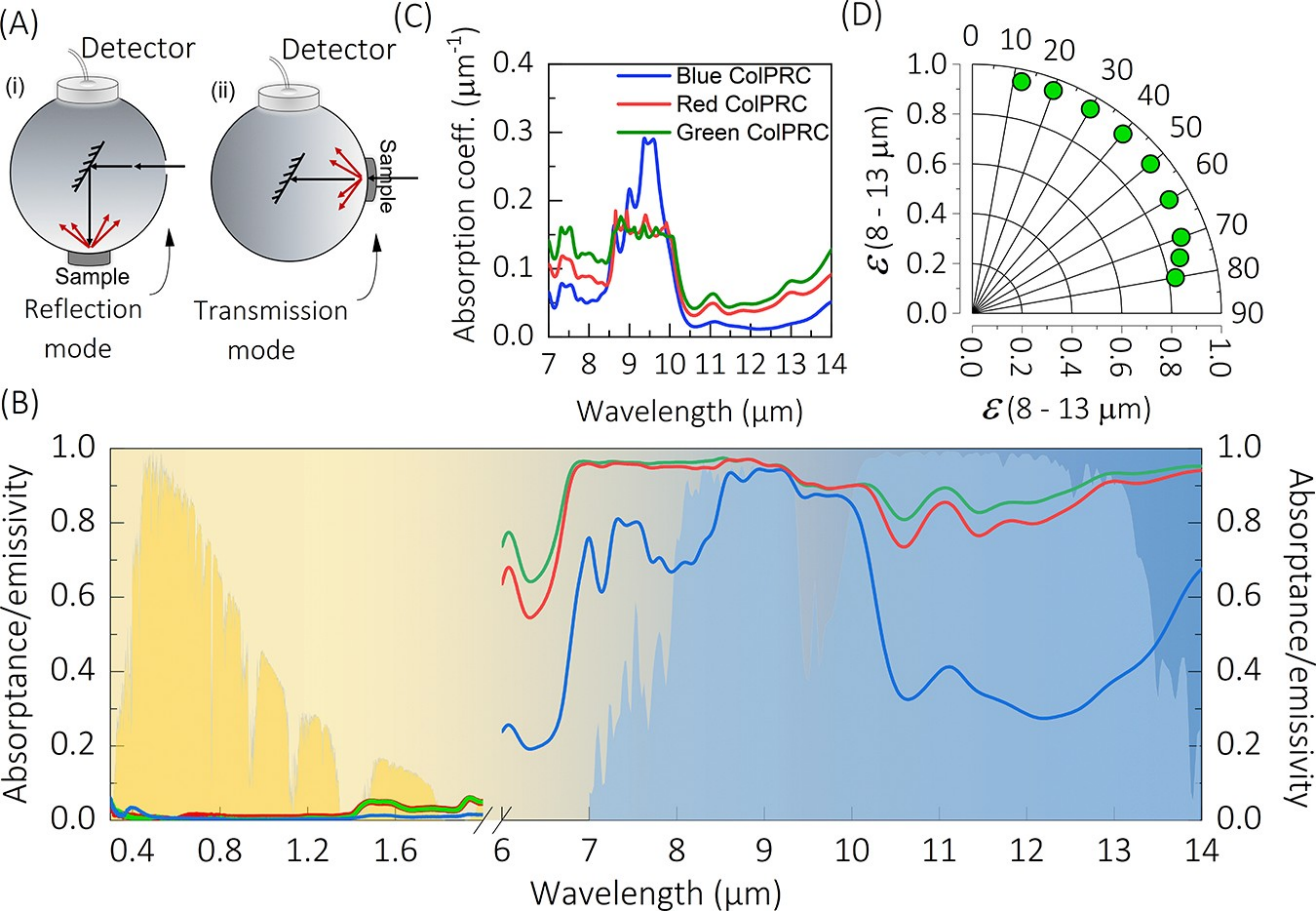
Solar and IR absorptance/emissivity of CNC ColPRCs. (A) Schematic diagrams of experimental integrating sphere setups for measurements of (i) IR reflectance and (ii) transmittance used to determine the absorptance/emissivity. (B) Absorptance spectra for red, green, and violet-blue colored CNC ColPRCs together with the AM1.5 solar spectrum in the left part (dark yellow area) in arbitrary units and the atmospheric transmittance (blue area) in the right part. (C) Absorption coefficients of the differently colored ColPRCs to account for different film thicknesses. The thickness was determined to be lower for the violet-blue ColPRC (around 23 μm) compared with the other two samples (around 47 μm). (D) Average measured emissivity (ε) at different angles of a green ColPRC.

We will now investigate the absorptance of the CNC ColPRC films in both the solar and infrared spectral ranges. For efficient daytime PRC, the films should provide low solar absorption but strong IR absorption (which equals emissivity (ε) as per Kirchhoff’s law of thermal radiation).^34,35^ As depicted in Figure 3a, we determined the absorptance from the (i) reflectance *R*(λ) and (ii) transmittance *T*(λ) using integrating sphere measurements to account for nonspecular backward and forward scattering by the films (see further details in the Methods section). Figure 3b shows the emissivity/absorptance of violet-blue, green and red ColPRCs films (in respectively colored solid lines) along with the air mass 1.5 (AM1.5) solar spectrum in yellow and IR transparency of the atmosphere in violet-blue. All three CNC ColPRCs showed very low absorption (<5%) in the whole range from 0.3 to 1.3 μm, which is promising to avoid solar heating. Interestingly, the presumed increased light interaction due to the Bragg reflection results only in a maximum few percent increase in absorption (see Figure S2). The absorption increases in the UV and near-IR ranges, but the solar irradiance is also lower in these ranges (compare with the yellow shaded solar spectrum in Figure 3b). Hence, our ColPRCs provide low solar absorption combined with controllable structural colors.

In the IR region, all three CNC ColPRCs show high absorptance/emissivity, with values above 80% at 10 μm for all samples. This is attributed to the inherent strong IR absorption of cellulose via vibrational resonances.^36,37^ The violet-blue ColPRC showed lower absorptance than the red and green samples, which likely is related to differences in film thickness. Indeed, the violet-blue film was found thinner when measuring with a high-accuracy digital micrometer. We therefore estimated the experimental intrinsic absorption coefficient, α (absorption per unit thickness for a given wavelength) using^38^
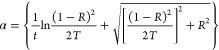
2where we set *t*, *R*, and *T* as the film thickness, total reflectance, and total transmittance, respectively. Figure 3c presents the results, suggesting that part of the differences in absorptance between the samples can be attributed to thickness variations. The violet-blue ColPRC sample shows a somewhat higher α around 9–10 μm, while the red and green ColPRCs provide a higher absorption coefficient in most of the remaining broad spectral regions. We also measured the emissivity as a function of angle for a green ColPRC (see Figure S3 for similar measurements for violet-blue and red ColPRCs). As shown in Figure 3d, the emissivity (presented as the average in the range 8 μm–13 μm) of the ColPRC is high for low angles (close to the normal) and remains high up to high angles of 60°–70°, after which it decreases at even more oblique angles. All combined measurements, across the solar and thermal emissivity spectral ranges, suggest that the structurally colored CNC films provide highly suitable properties for transreflective ColPRC, which we investigate next.

**Figure 4:**
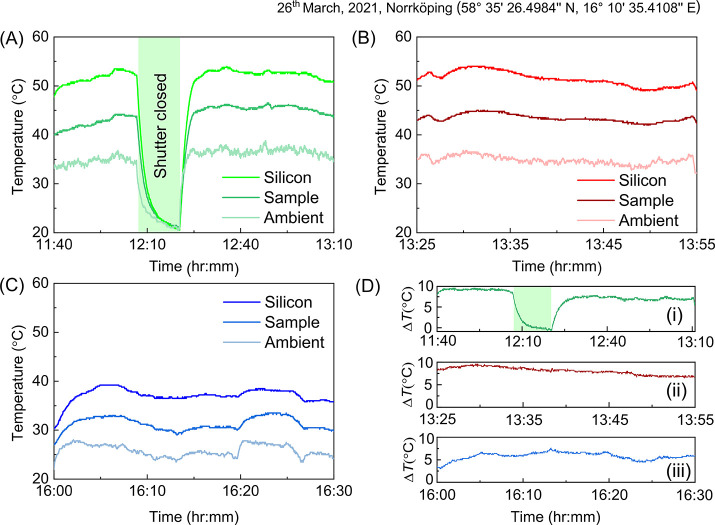
Rooftop outdoor measurements of the photonic radiative coolers’ performance on March 26, 2021 in Norrköping. Temperature evolution of (A) a green ColPRC, (B) a red ColPRC, and (C) a violet-blue ColPRC along with ambient and silicon substrate while exposed to the sun and the sky. The container was closed by a shutter during the green shaded area in (A). (D) Reduction in temperature from the noncoated silicon sample (Δ*T*) over time for (i) the green ColPRC, (ii) the red ColPRC, and (iii) the violet-blue ColPRC.

We evaluate the CNC films for ColPRCs by using them as coatings on silicon samples and monitoring the temperatures of the samples while exposing them to the clear sky and sunlight during daytime. For each measurement, a CNC ColPRC-coated silicon sample and a noncoated reference silicon sample were placed in the measurement chamber, close to each other to ensure nearly identical environment and conditions (see details in Figure S4). Figure 4a–c shows the temperature evolution of violet-blue, green, and red CNC ColPRCs samples, respectively, together with the results for the noncoated silicon reference substrate and the ambient temperature inside the measurement container. As expected, the temperature of the bare silicon substrate was consistently much higher (up to around 20 °C higher) than the ambient temperature inside the measurement chamber. Importantly, all three CNC ColPRCs efficiently reduced the sample temperature compared with the noncoated samples, by on average ∼9 °C for the green and red ColPRCs and ∼6 °C for the violet-blue ColPRC. This is also evident from Figure 4d, which presents the difference in temperature between the noncoated sample and the coated samples (Δ*T*). The temperature reduction is attributed to lowered solar heating due to wavelength-selective reflection and to PRC via thermal radiation through the atmospheric window. Indeed, covering the setup with a metal foil to prevent both solar heating and PRC (between 12:00 and 12:25 in Figure 4a) led to sample temperatures that approached that of the ambient. To separately investigate the contribution of PRC to the temperature reduction, we shaded the measurement container from direct solar exposure, while keeping it open for thermal radiation to the sky. As shown in Figure S5 for a green sample, the CNC ColPRC coating managed to reduce the sample temperature to below that of the ambient, confirming that PRC contributes to the reduction in temperature. From Figure 4, we also note that the red and green ColPRCs showed a larger reduction in temperature compared to the violet-blue CNC sample. This may in part be due to stronger solar irradiance in the green and red wavelength regions, leading to more effective suppression of solar heating for the green and red samples due to their higher reflection in those regions. In that respect, we note that measurements using a noncolored CNC film as coating showed less effective reduction in temperature compared with all three transreflective ColPRCs (Figure S6). Another contribution to the difference in reduction in temperature between the ColPRCs could be lower cooling performance for the violet-blue ColPRC film due to its lower thermal emissivity (Figure 3b). Furthermore, we note that also the absolute temperatures were lower for the measurement with the violet-blue sample, indicating variations also of external factors like outdoor temperature or solar irradiance.

## Conclusion

We have experimentally demonstrated structurally colored photonic cellulose films for transreflective colored radiative cooling. Our CNC ColPRC films do not require any complicated or expensive fabrication protocols and only necessitates self-assembly of a cellulose nanocrystal dispersion together with varied amount of glucose to tune the coloration. In the visible region, the photonic cellulose films act as wavelength-selective reflectors, while the visible absorption remains minimal. High thermal emissivity combined with low visible absorption make the CNC ColPRCs suitable for radiative cooling, also in sunny conditions during daytime. As coatings on silicon substrates, the CNC ColPRCs could lower the substrate temperature by as much as 9 °C. Although exact values are affected by variations in measurement and weather conditions, we note that this reduction in temperature when applied as coating on silicon is comparable to that of previous colored transparent PRCs based on self-assembled silica nanospheres.^39^ We found that the temperature reduction for our concept was due to a combination of PRC and suppressed solar absorption due to the wavelength-selective reflection of the colored CNC films. Our study provides a scalable and sustainable method for transreflective PRC for cooling outdoor structures and devices with functional or aesthetic considerations. The general concept may further be adopted to ColPRCs with wavelength-selective reflection/transmission that is specifically tailored to a certain device, such as to the absorption band of a photovoltaic cell.

## Methods

### Preparation of CNCs and Fabrication of CNC/GLU Films

Preparation of CNCs and fabrication of CNC/GLU ColPRC was obtained following the procedure described in detail in a previous publication.^30^ In brief, microcrystalline cellulose (Avicel PH-200) was hydrolyzed using sulfuric acid, followed by iterative centrifugation/washing steps, and dialysis of the supernatant (rich in CNCs) obtained after the third centrifugation step. The obtained CNC suspension was filtered with Whatman filter paper and concentrated to 2.5 wt %. To obtain CNC/GLU, 1 wt % glucose solution was added to the 2.5 wt % CNC aqueous suspension. Various colors ColPRC were achieved by varying the composition of the CNC/GLU suspension. The noncolored CNC film was obtained by using 0% GLU. The freestanding ColPRC films were made by solvent-casting the CNC/GLU suspension in a polystyrene Petri dish coated with Teflon coating (BytacTM, Saint-Gobain) and dried at ambient conditions for 2–3 days.

### Characterization

Reflectance, transmittance, and absorptance of samples were determined using spectral directional hemispherical reflectance (DHR) and directional hemispherical transmittance (DHT). Two different spectrometers were used to cover the wavelength regions from the UV to the far IR: A Cary 5000 in the region 250–2500 nm and a Bruker Vertex 70 FTIR spectrometer for the region 2–33 μm. Both instruments were equipped with integrating spheres illuminating the sample at an angle of incidence (θ_i_) of 8° and 9°, for the Cary and Bruker spectrometers, respectively. A DRA-2500 integrating sphere from Labsphere was used for the Cary spectrophotometer, and a Labsphere A562 was used for the Bruker FTIR. The following detectors were used for the different spectral ranges: R928 PMT for UV–vis, a cooled PbS for NIR up to 2500 nm, and a DTGS detector in the IR.

For reflectance measurements, both instruments make use of calibrated reflectance standards (Spectralon and Infragold calibrated standards from Labsphere). For the Cary instrument, the reflectance standard was used to collect the baseline, which was used when calculating the sample DHR. The integrating sphere of the Bruker FTIR makes use of an absolute reflection method using the interior wall of the sphere for the baseline measurement, but where the results of the DHR are corrected by a factor obtained from measurements on different calibrated reflectance standards to ensure accurate measurement results. The spectral absorptance was then obtained from the spectral reflectance and transmittance via:
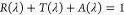
3where *R*, *T*, and *A* are in the range from 0 to 1 (or 0% to 100%). We note here that our measurements (DHR and DHT) account for diffusive reflection and transmission and not only specular reflection and collimated transmission. By Kirchhoff’s law of radiation, the emissivity at any given wavelength (ε(λ)) for a surface in thermal equilibrium is then given by:
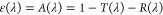
4

### Radiative Cooling Measurements

Figure S4a,b shows a schematic illustration and a photograph illustration of the radiative cooling measurement setup. The setup consists of one chamber with bottom and surfaces covered with reflective metalized Mylar sheets. Samples and ambient reference thermocouple were placed close to the middle of the temperature measurement box of around 40 cm × 30 cm × 15 cm. Foam blocks wrapped with reflective metalized Mylar sheets were placed inside the chamber. The silicon substrate was then placed onto these blocks representing objects to be cooled. The ColPRCs were then used as colored coatings on these silicon substrates while monitoring their temperature during their exposure to the sun and open sky. A 12 μm polyethylene sheet was used to cover the measurement box in order to serve as a wind shield and protect from moisture. One thermocouple was present freely in the box to measure the air temperature as the ambient temperature. Each cooler was stuck to a silicon wafer using stripes of double-sided adhesive tape at the edges to ensure that most of the bottom surface area of the cooler was in direct contact with the silicon wafer. Real-time temperature values of the thermocouples were recorded using a LabVIEW program interfaced by an Arduino processor.

The cooling performance (Figure 4) was measured on an outdoor rooftop at Linköping university, campus Norrköping (58° 35′ 26.4984″ N, 16° 10′ 35.4108″ E) on March 26, 2021. During the afternoon of March 26, 2021, the average outdoor temperature, wind speed, and relative humidity in the city were ∼11 °C, ∼ 2 m/s, and ∼68%, respectively. The cooling measurement in Figure S5 was performed on the morning of April 14, 2021 when the average outdoor temperature, wind speed, and relative humidity were ∼8 °C, ∼3 m/s, and ∼35%, respectively.

## Supporting Information Available

Scanning electron microscope images of cross sections of ColPRC films; experimentally measured absorption spectra of ColPRCs; angle-dependent emissivity of Violet-blue and Red ColPRC; schematic cross-section of a radiative cooler setup and image of the real-time temperature measurement setup with a green ColPRC cooler and bare Si wafer placed in it; real-time temperature and temperature difference measurement on the green ColPRC under no direct solar irradiance; and measurement for a noncolored CNC sample on silicon (PDF)

## References

[ref1] Fan S. Thermal photonics and energy applications. Joule 2017, 1 (2), 264–273. 10.1016/j.joule.2017.07.012.

[ref2] Hossain M. M.; Gu M. Radiative cooling: principles, progress, and potentials. Advanced Science 2016, 3 (7), 1500360 10.1002/advs.201500360. 27812478 PMC5067572

[ref3] Li W.; Fan S. Radiative cooling: harvesting the coldness of the universe. Optics and Photonics News 2019, 30 (11), 32–39. 10.1364/OPN.30.11.000032.

[ref4] Sun X.; Sun Y.; Zhou Z.; Alam M. A.; Bermel P. Radiative sky cooling: fundamental physics, materials, structures, and applications. Nanophotonics 2017, 6 (5), 997–1015. 10.1515/nanoph-2017-0020.

[ref5] Yin X.; Yang R.; Tan G.; Fan S. Terrestrial radiative cooling: Using the cold universe as a renewable and sustainable energy source. Science 2020, 370 (6518), 786–791. 10.1126/science.abb0971. 33184205

[ref6] Zhang Y.; Chen X.; Cai B.; Luan H.; Zhang Q.; Gu M. Photonics Empowered Passive Radiative Cooling. Advanced Photonics Research 2021, 2 (4), 2000106 10.1002/adpr.202000106.

[ref7] Raman A. P.; Anoma M. A.; Zhu L.; Rephaeli E.; Fan S. Passive radiative cooling below ambient air temperature under direct sunlight. Nature 2014, 515 (7528), 540–544. 10.1038/nature13883. 25428501

[ref8] Chen Y.; Dang B.; Fu J.; Wang C.; Li C.; Sun Q.; Li H. Cellulose-Based Hybrid Structural Material for Radiative Cooling. Nano Lett. 2021, 21, 397–404. 10.1021/acs.nanolett.0c03738. 33301320

[ref9] Tian Y.; Shao H.; Liu X.; Chen F.; Li Y.; Tang C.; Zheng Y. Superhydrophobic and Recyclable Cellulose-Fiber-Based Composites for High-Efficiency Passive Radiative Cooling. ACS Appl. Mater. Interfaces 2021, 13, 22521–22530. 10.1021/acsami.1c04046. 33950669

[ref10] Gamage S.; Kang E. S.; Åkerlind C.; Sardar S.; Edberg J.; Kariis H.; Ederth T.; Berggren M.; Jonsson M. P. Transparent nanocellulose metamaterial enables controlled optical diffusion and radiative cooling. Journal of Materials Chemistry C 2020, 8 (34), 11687–11694. 10.1039/D0TC01226B.

[ref11] Gamage S.; Banerjee D.; Alam Md. M.; Hallberg T.; Akerlind C.; Sultana A.; Shanker R.; Berggren M.; Crispin X.; Kariis H.; Zhao D.; Jonsson M. P. Reflective and transparent cellulose-based passive radiative coolers. Cellulose 2021, 28, 9383–9393. 10.1007/s10570-021-04112-1.

[ref12] Li W.; Shi Y.; Chen K.; Zhu L.; Fan S. A comprehensive photonic approach for solar cell cooling. Acs Photonics 2017, 4 (4), 774–782. 10.1021/acsphotonics.7b00089.

[ref13] Zhu L.; Raman A. P.; Fan S. Radiative cooling of solar absorbers using a visibly transparent photonic crystal thermal blackbody. Proc. Natl. Acad. Sci. U. S. A. 2015, 112 (40), 12282–12287. 10.1073/pnas.1509453112. 26392542 PMC4603484

[ref14] Zhu L.; Raman A.; Fan S. Color-preserving daytime radiative cooling. Appl. Phys. Lett. 2013, 103 (22), 223902 10.1063/1.4835995.

[ref15] Cai L.; Peng Y.; Xu J.; Zhou C.; Zhou C.; Wu P.; Lin D.; Fan S.; Cui Y. Temperature regulation in colored infrared-transparent polyethylene textiles. Joule 2019, 3 (6), 1478–1486. 10.1016/j.joule.2019.03.015.

[ref16] Li W.; Shi Y.; Chen Z.; Fan S. Photonic thermal management of coloured objects. Nat. Commun. 2018, 9 (1), 4240 10.1038/s41467-018-06535-0. 30315155 PMC6185958

[ref17] Heo S.-Y.; Lee G. J.; Kim Y. J.; Ishii S.; Kim M. S.; Seok T. J.; Lee B. J.; Lee H.; Song Y. M. A Janus emitter for passive heat release from enclosures. Science advances 2020, 6 (36), eabb1906 10.1126/sciadv.abb1906. 32917610 PMC7473666

[ref18] Blandre E.; Yalçin R. A.; Joulain K.; Drévillon J. Microstructured surfaces for colored and non-colored sky radiative cooling. Opt. Express 2020, 28 (20), 29703–29713. 10.1364/OE.401368. 33114863

[ref19] Chen Y.; Mandal J.; Li W.; Smith-Washington A.; Tsai C.-C.; Huang W.; Shrestha S.; Yu N.; Han R. P.; Cao A.; et al. Colored and paintable bilayer coatings with high solar-infrared reflectance for efficient cooling. Science Advances 2020, 6 (17), eaaz5413 10.1126/sciadv.aaz5413. 32426464 PMC7182418

[ref20] Levinson R.; Akbari H.; Berdahl P.; Wood K.; Skilton W.; Petersheim J. A novel technique for the production of cool colored concrete tile and asphalt shingle roofing products. Sol. Energy Mater. Sol. Cells 2010, 94 (6), 946–954. 10.1016/j.solmat.2009.12.012.

[ref21] Yalçın R. A.; Blandre E.; Joulain K.; Drévillon J. Colored Radiative Cooling Coatings with Nanoparticles. ACS Photonics 2020, 7 (5), 1312–1322. 10.1021/acsphotonics.0c00513.

[ref22] Kim M.; Lee D.; Son S.; Yang Y.; Lee H.; Rho J. Visibly Transparent Radiative Cooler under Direct Sunlight. Advanced Optical Materials 2021, 9, 2002226 10.1002/adom.202002226.

[ref23] Habibi Y.; Lucia L. A.; Rojas O. J. Cellulose nanocrystals: chemistry, self-assembly, and applications. Chem. Rev. 2010, 110 (6), 3479–3500. 10.1021/cr900339w. 20201500

[ref24] Moriana R.; Vilaplana F.; Ek M. Cellulose nanocrystals from forest residues as reinforcing agents for composites: A study from macro-to nano-dimensions. Carbohydr. Polym. 2016, 139, 139–149. 10.1016/j.carbpol.2015.12.020. 26794957

[ref25] Vincent S.; Kandasubramanian B. Cellulose nanocrystals from agricultural resources: Extraction and functionalisation. Eur. Polym. J. 2021, 160, 110789 10.1016/j.eurpolymj.2021.110789.

[ref26] Guidetti G.; Atifi S.; Vignolini S.; Hamad W. Y. Flexible photonic cellulose nanocrystal films. Adv. Mater. 2016, 28 (45), 10042–10047. 10.1002/adma.201603386. 27748533 PMC5495155

[ref27] Kelly J. A.; Giese M.; Shopsowitz K. E.; Hamad W. Y.; MacLachlan M. J. The development of chiral nematic mesoporous materials. Accounts of chemical research 2014, 47 (4), 1088–1096. 10.1021/ar400243m. 24694253

[ref28] Kose O.; Tran A.; Lewis L.; Hamad W. Y.; MacLachlan M. J. Unwinding a spiral of cellulose nanocrystals for stimuli-responsive stretchable optics. Nat. Commun. 2019, 10 (1), 510 10.1038/s41467-019-08351-6. 30705267 PMC6355765

[ref29] Tran A.; Boott C. E.; MacLachlan M. J. Understanding the Self-Assembly of Cellulose Nanocrystals—Toward Chiral Photonic Materials. Adv. Mater. 2020, 32 (41), 1905876 10.1002/adma.201905876. 32009259

[ref30] Anusuyadevi R. P.; Shanker R.; Cui Y.; Riazanova A. V.; Järn M.; Jonsson M. P.; Svagen A. J. Photoresponsive and Polarisation-sensitive Structural Colors from Cellulose/Liquid Crystal Nanophotonic structures. Adv. Mater. 2021, 33 (36), 2101519 10.1002/adma.202101519. PMC11468386 34313346

[ref31] Frka-Petesic B.; Kelly J. A.; Jacucci G.; Guidetti G.; Kamita G.; Crossette N. P.; Hamad W. Y.; MacLachlan M. J.; Vignolini S. Retrieving the Coassembly Pathway of Composite Cellulose Nanocrystal Photonic Films from their Angular Optical Response. Adv. Mater. 2020, 32 (19), 1906889 10.1002/adma.201906889. PMC7116217 32249481

[ref32] Li T.; Zhai Y.; He S.; Gan W.; Wei Z.; Heidarinejad M.; Dalgo D.; Mi R.; Zhao X.; Song J.; et al. A radiative cooling structural material. Science 2019, 364 (6442), 760–763. 10.1126/science.aau9101. 31123132

[ref33] Almeida A. P.; Canejo J. P.; Fernandes S. N.; Echeverria C.; Almeida P. L.; Godinho M. H. Cellulose-based biomimetics and their applications. Adv. Mater. 2018, 30 (19), 1703655 10.1002/adma.201703655. 29333680

[ref34] Le Gall J.; Olivier M.; Greffet J.-J. Experimental and theoretical study of reflection and coherent thermal emissionby a SiC grating supporting a surface-phonon polariton. Phys. Rev. B 1997, 55 (15), 10105 10.1103/PhysRevB.55.10105.

[ref35] Greffet J.-J.; Nieto-Vesperinas M. Field theory for generalized bidirectional reflectivity: derivation of Helmholtz’s reciprocity principle and Kirchhoff’s law. JOSA A 1998, 15 (10), 2735–2744. 10.1364/JOSAA.15.002735.

[ref36] Tshabalala M. A. Surface characterization, Handbook of wood chemistry and wood composites, 2nd ed.; CRC Press: Boca Raton, FL, 2012; pp 217–252.

[ref37] Sofla M. R. K.; Brown R.; Tsuzuki T.; Rainey T. A comparison of cellulose nanocrystals and cellulose nanofibres extracted from bagasse using acid and ball milling methods. Adv. Nat. Sci: Nanosci. Nanotechnol. 2016, 7 (3), 035004 10.1088/2043-6262/7/3/035004.

[ref38] Kranjčec M.; Studenyak I.; Kurik M. Urbach rule and disordering processes in Cu6P (S1– xSex) 5Br1– yIy superionic conductors. J. Phys. Chem. Solids 2006, 67 (4), 807–817. 10.1016/j.jpcs.2005.10.184.

[ref39] Kim H. H.; Im E.; Lee S. Colloidal photonic assemblies for colorful radiative cooling. Langmuir 2020, 36 (23), 6589–6596. 10.1021/acs.langmuir.0c00051. 32370514

