# Supplementary material for: Structurally Colored Cellulose Nanocrystal Films as Transreflective Radiative Coolers
Source: ACS Nano. 2022 Jun 6;16(7):10156–62. doi: 10.1021/acsnano.1c10959 (PMC9331159; doi:10.1021/acsnano.1c10959)
Supplement: Supplementary file 1 — nn1c10959_si_001.pdf [file nn1c10959_si_001.pdf]

Supporting Information

Structurally Colored Cellulose Nanocrystal films as Trans-Reflective Radiative Coolers

Ravi Shanker[†,‡], Prasaanth Ravi Anusuyadevi[§&], Sampath Gamage[†,‡], Tomas Hallberg[#], Hans Kariis[#], Debashree Banerjee[†], Anna J. Svagan[§] and Magnus P. Jonsson[†,‡,*]

[†‡]Laboratory of Organic Electronics, Department of Science and Technology, Linköping University, SE-601 74 Norrköping, Sweden

[‡]Wallenberg Wood Science Center, Linköping University, SE-601 74 Norrköping, Sweden

[§]Royal Institute of Technology (KTH), Dept. of Fibre and Polymer Technology, SE-100 44 Stockholm, Sweden

[#]FOI-Swedish Defense Research Agency, Department of Electro-Optical systems, 583 30 Linköping, Sweden

[&]Department of Chemical Engineering, M S Ramaiah Institute of Technology, 560054 Bangalore, Karnataka, India

*Corresponding author: magnus.jonsson@liu.se

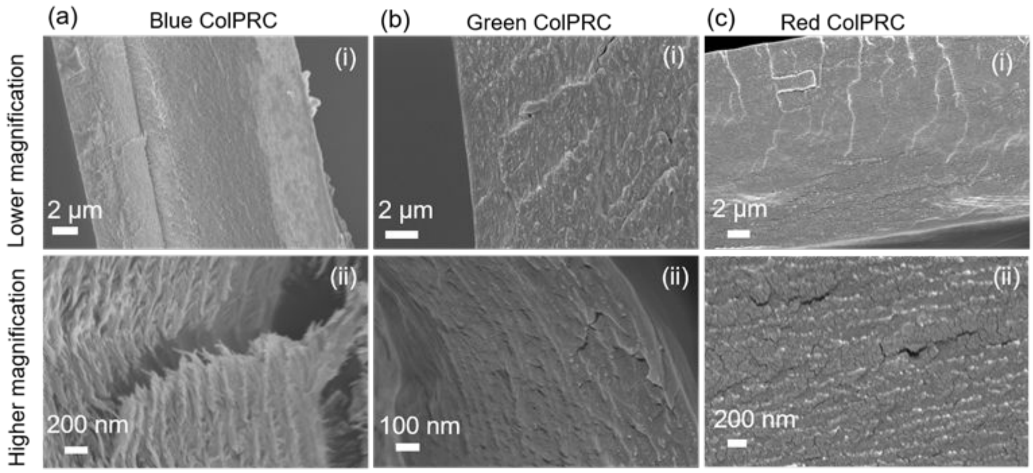

Figure. S1. Scanning electron microscope (SEM) images of cross-sections of ColPRC films, (a) violet-blue ColPRC, (b) green ColPRC, (c) red ColPRC showing parallel arc like morphology of planar ordering, constant pitch and spatially varying pitch within a planar domain, and tilted domains next to planar ones. At low magnification, SEM images indicate that the layers are parallel to each other while at higher magnification the images reveal pitch length and the preferential orientation of CNCs.

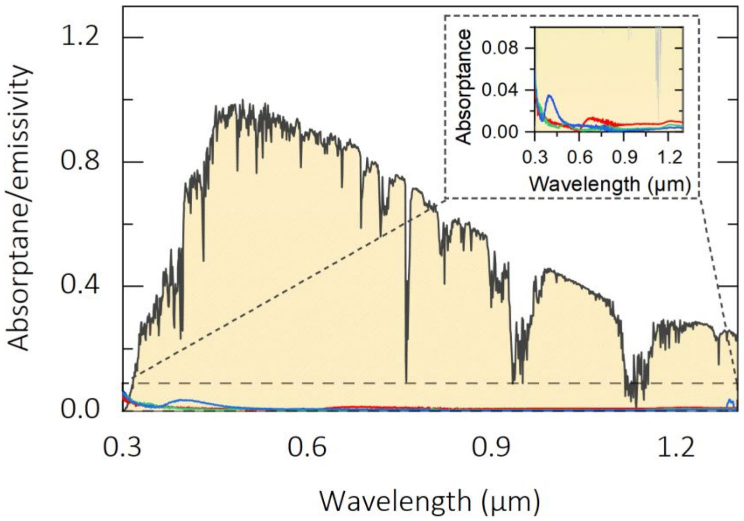

Figure. S2. Experimentally measured absorption spectra of ColPRCs. The inset shows that all three CNC ColPRCs showed very low absorption (<5%) in the whole range from 0.3 to

1.3 μm. The shaded region (yellow color) represents the normalized terrestrial solar spectral irradiance spectrum at reference AM 1.5.

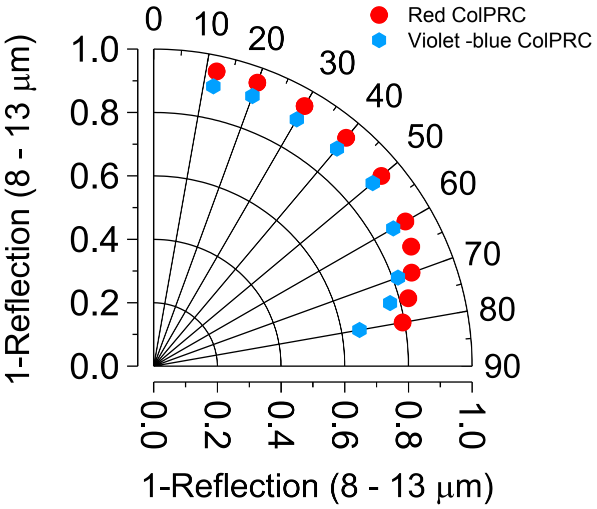

Figure. S3. Angle dependent emissivity of ColPRC. (a) Angle dependent emissivity of Violet-blue and Red ColPRC measured in (1-reflection) mode.

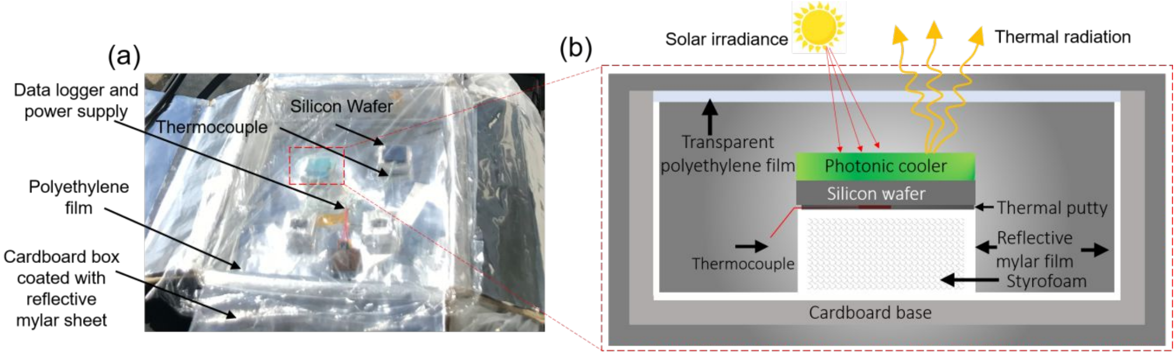

Figure. S4. Digital photograph and schematic illustration of the radiative cooling measurement setup. (a) Photograph of the real-time temperature measurement setup, with a green ColPRC cooler and a bare Si sample placed in it. (b) Schematic cross-section of the setup illustrating the sample with the ColPRC coating, position of thermocouple, and other details.

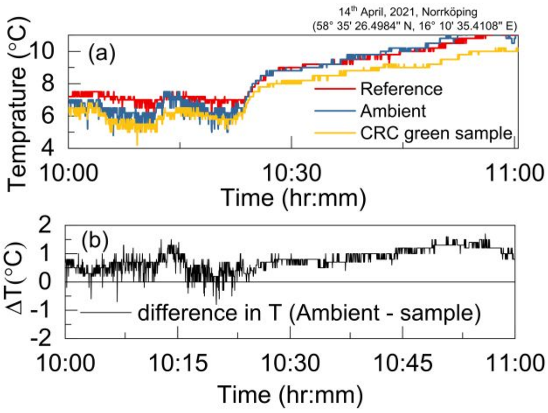

Figure. S5. Real-time temperature and temperature difference measurement for the green ColPRC under no direct solar irradiance, on April 14, 2021 in Norrköping. The green ColPRC achieved temperature differentials of ~ 1°C lower than the ambient temperature inside the measurement chamber.

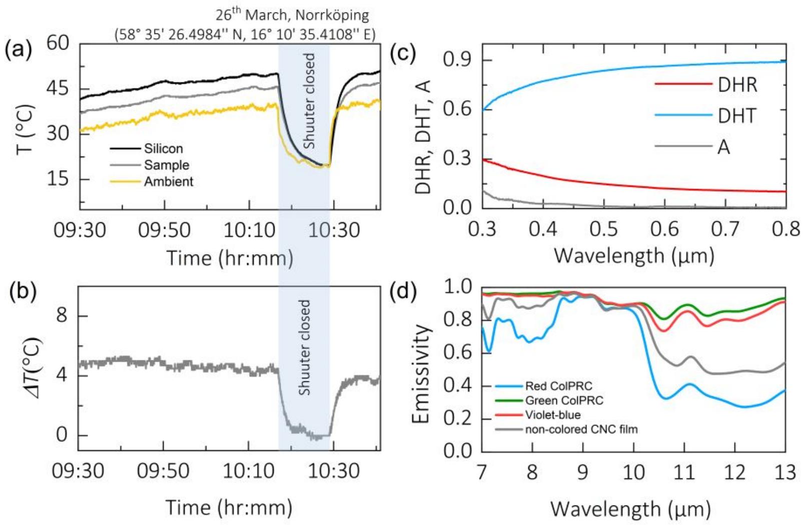

Figure. S6. (a) Cooling performance measurement for a non-colored CNC film on silicon (Sample) along with the temperature of a non-coated silicon substrate and the ambient temperature in the chamber, while exposed to sun and sky on 26th March 2021 in Norrköping. (b) The non-colored CNC film showed a temperature of ~ 5°C lower than the non-coated silicon sample (ΔT). The setup was closed by a shutter during the shaded area. (c) Reflectance (DHR), transmittance (DHT), and absorptance (A) for the non-colored CNC coating. (d) Emissivity (i.e. absorptance) of non-colored CNC coating along with colored coolers.
